# *Bcl10* is not a target for frequent mutation in human carcinomas

**DOI:** 10.1038/sj.bjc.6690564

**Published:** 1999-07

**Authors:** A R Lambers, C Gumbs, S Ali, J R Marks, J D Iglehart, A Berchuck, P A Futreal

**Affiliations:** Departments of Surgery, Obstetrics/Gynecology – Division of Gynecologic Oncology and Genetics, Duke University Medical Center, Durham, NC 27710, USA

## Abstract

The recently described *Bcl10* gene has been suggested to be a major target gene for inactivation in a variety of human cancers. In order to further evaluate the role of this gene in human adult malignancies, we have analysed a series of carcinomas for mutations in the *Bcl10* gene. We have screened a panel of 174 carcinoma samples in total, comprised of 47 breast, 36 epithelial ovarian, 36 endometrial, 12 cervical, 23 colorectal and 20 head/neck carcinomas, all unselected for grade or stage. This panel reflects, in part, tumours reported to have involvement of the 1p22 region of chromosome 1, the region harbouring the *Bcl10* gene. No deleterious mutations were detected in any of the samples analysed, strongly suggesting that *Bcl10* is not a common target for inactivation in adult malignancies and that BCL10 is not the gene targeted for frequent inactivation at 1p22. © 1999 Cancer Research Campaign


*British Journal of Cancer* (1999) **80**(10), 1575–1576
© 1999 Cancer Research Campaign
Article no. bjoc.1999.0564

### *Bcl10* is not a target for frequent mutation in human carcinomas

**AR Lambers[1], C Gumbs[1], S Ali[1], JR Marks[1], JD Iglehart[1], A Berchuck[2] and PA Futreal[1,2,3]**

Departments of [1]Surgery, [2]Obstetrics/Gynecology – Division of Gynecologic Oncology and [3]Genetics. Duke University Medical Center, Durham, NC 27710, USA

**Summary** The recently described *Bcl10* gene has been suggested to be a major target gene for inactivation in a variety of human cancers. In order to further evaluate the role of this gene in human adult malignancies, we have analysed a series of carcinomas for mutations in the *Bcl10* gene. We have screened a panel of 174 carcinoma samples in total, comprised of 47 breast, 36 epithelial ovarian, 36 endometrial, 12 cervical, 23 colorectal and 20 head/neck carcinomas, all unselected for grade or stage. This panel reflects, in part, tumours reported to have involvement of the 1p22 region of chromosome 1, the region harbouring the *Bcl10* gene. No deleterious mutations were detected in any of the samples analysed, strongly suggesting that *Bcl10* is not a common target for inactivation in adult malignancies and that BCL10 is not the gene targeted for frequent inactivation at 1p22.

The *Bcl10* gene, mapping to human chromosome 1p22, has recently been shown to be disrupted by translocations in MALT lymphomas carrying a t(1;14)(p22;q32) translocation (Willis et al, 1999). These translocations map upstream of the coding sequences of *Bcl10* and are accompanied by protein truncating mutations within the coding sequence itself. Wild-type *Bcl10* appears to function as regulatory molecule in pro-apoptotic pathways, whereas mutant *Bcl10* alleles have been reported to have transforming activity in cell culture (Willis et al, 1999). In addition to its apparent role in MALT lymphoma, it has been reported that *Bcl10* is frequently mutated in multiple tumour types and that inactivation of the *Bcl10* gene may be a common facet in the pathogenesis of human cancers (Willis et al, 1999). In order to address more critically the potential role of *Bcl10* in human cancers, we have studied a series of adult carcinomas for mutations in this locus. Specifically, a panel of 174 cancers targeted primarily to those tumours reported to have frequent involvement of the 1p22 region from prior molecular genetic studies was examined (Jin et al, 1990; Bardi et al, 1995; Hoggard et al, 1995; Mertens et al, 1997; Tsukamoto et al, 1998; Tong and Futreal, manuscript in preparation). The screening panel was comprised of breast, ovarian, endometrial, cervical, colorectal and head/neck carcinomas (Table 1).

### MATERIALS AND METHODS

Genomic DNA was extracted from frozen tumours using the PureGene kit (Gentra Systems Inc., Minneapolis, MN, USA). Screening for mutations was carried out using single-strand conformation analysis (SSCA) followed by direct sequencing. The polymerase chain reaction (PCR) amplimers used in the analysis were identical to those from Willis et al (1999) and allowed for screening of both coding sequences and intron–exon borders of the three coding exons of *Bcl10*. Standard reaction conditions (annealing at 55°C) were used with radioactive label incorporation as described previously (Phelan et al, 1996), except for exon 1 for which a touchdown (annealing from 65°C to 55°C, stepdown of 0.5°C) reaction profile was used. PCR products were denatured in formamide-loading buffer, cooled rapidly and loaded onto 0.5 × MDE gels which were run overnight at 5–8 W. Gels were dried and autoradiographed. Any aberrantly migrating bands were excised from the gels and direct sequenced using the PCR amplimers on an ABI-Prizm 377 sequencer as previously described (Phelan et al., 1996).

### RESULTS AND DISCUSSION

No mutations were detected in exons 2 and 3 in any of the samples analysed. Analysis of exon 1 revealed what was most likely a complex polymorphism on SSCA analysis given the frequency of aberrant shifts. Direct sequencing of a subset of the prominent shifts detected the following two polymorphisms: G13T/Ala5Ser and G24C/Leu8Leu. Given the common nature of these changes and the fact that one encodes a silent amino change, these most likely represent benign polymorphisms and not deleterious changes.

Our data do not support the notion that *Bcl10* is a frequent target of inactivation in human cancer, or that it is unusually susceptible to mutation as has been suggested. Our analysis was restricted to genomic DNAs derived from primary tumours as opposed to

**Table 1** Carcinoma panel screened for *Bcl10* mutations

| Tumour type | Number examined |
|---|---|
| Breast cancer | 47 |
| Epithelial ovarian cancer | 36 |
| Endometrial cancer | 36 |
| Cervical cancer | 12 |
| Colorectal cancer | 23 |
| Head/neck cancer | 20 |
| | Total: 174 |

*Received 4 May 1999*
*Accepted 4 May 1999*

*Correspondence to:* PA Futreal, PO Box 2611, Duke University Medical Center, Durham, NC 27710, USA

analysis of cell lines in the original study, and thus the differences reported here may be, in part, attributable to acquisition of mutations in cell culture. Additionally, it is not clear what fraction of mutations detected previously were found only in cDNA, another factor that may influence the rate of mutation seen, as alterations in transcripts derived from tumours have been reported that do not correspond to bona fide genomic alterations of the target gene (Carney et al, 1998). Our data would further suggest that *Bcl10* is not the critical 1p22 gene targeted for inactivation in multiple tumour types.

### ACKNOWLEDGEMENTS

We thank Dr Doug Tyler and Dr WJ Richtsmeier for providing colorectal and head/neck tumour samples and Julia Lancaster for sample databasing. This work was supported in part by the NCI/Duke SPORE in breast cancer (P50-CA68438) and the Mary Kay Ash Foundation.

### REFERENCES

Bardi G, Sukhikh T, Pandis N, Fenger C, Kronborg O and Heim S (1995) Karyotypic characterization of colorectal adenocarcinomas. *Genes Chromosomes Cancer* **12**: 97–109

Carney ME, Maxwell GL, Lancaster JM, Gumbs C, Marks J, Berchuck A and Futreal PA (1998) Aberrant splicing of the TSG101 tumor suppressor gene in human breast and ovarian cancers. *J Soc Gynecol Invest* **5**: 281–285

Hoggard N, Brintnell B, Howell A, Weissenbach J and Varley J (1995) Allelic imbalance on chromosome 1 in human breast cancer II: microsatellite repeat analysis. *Genes Chromosomes Cancer* **12**: 24–31

Jin YS, Higashi K, Mandahl N, Heim S, Wennerberg J, Bjorklund A, Dictor M and Mitelman F (1990) Frequent rearrangement of chromosomal bands 1p22 and 11q13 in squamous cell carcinomas of the head and neck. *Genes Chromosomes Cancer* **2**: 198–204

Mertens F, Johansson B, Hoglund M and Mitelman F (1997) Chromosomal imbalance maps of malignant solid tumors: a cytogenetic survey of 3185 neoplasms. *Cancer Res* **57**: 2765–2780

Phelan CM, Lancaster JM, Tonin P, Gumbs C, Cochran C, Carter R, Ghadirian P, Perret C, Moslthi R, Dion F, Faucher MC, Dole K, Karimi S, Foulkes W, Lounis H, Warner E, Goss P, Anderson D, Larsson C, Narod SA and Futreal PA (1996) Mutation analysis of the BRCA2 gene in 49 site-specific breast cancer families. *Nat Genet* **13:** 120–122

Tsukamoto K, Ito N, Yoshimoto M, Kasumi F, Akiyama F, Sakamoto G, Nakamura Y and Emi M (1998) Allelic loss on chromosome 1p is associated with progression and lymph node metastasis of primary breast carcinoma. *Cancer* **82**: 317–322

Willis TG, Jadayel CM, Ming-Qing D, Peng H, Perry AR, Abdul-Rant M, Price H, Karran L, Majekodunmi O, Wlodarska I, Pan L, Cruok I, Hamondi RA, Isaacson PG and Dyer MJS (1999) Bcl10 is involved in the t(1;14)(p22;q32) of MALT B cell lymphoma and mutated in multiple tumour types. *Cell* **96**: 34–45

*© 1999 Cancer Research Campaign*
